# Capabilities of multi-pinhole SPECT with two stationary detectors for in vivo rat imaging

**DOI:** 10.1038/s41598-020-75696-0

**Published:** 2020-10-29

**Authors:** Jan P. Janssen, Jan V. Hoffmann, Takayuki Kanno, Naoko Nose, Jan-Peter Grunz, Masahisa Onoguchi, Xinyu Chen, Constantin Lapa, Andreas K. Buck, Takahiro Higuchi

**Affiliations:** 1 Department of Nuclear Medicine, University Hospital Würzburg, Oberdürrbacher Strasse 6, 97080 Würzburg, Germany; 2 Comprehensive Heart Failure Centre, University Hospital Würzburg, Würzburg, Germany; 3 Department of Quantum Medical Technology, Graduate School of Medical Sciences, Kanazawa University, Kanazawa, Japan; 4 Graduate School of Medicine, Dentistry and Pharmaceutical Sciences, Okayama University, Okayama, Japan; 5 Department of Diagnostic and Interventional Radiology, University Hospital Würzburg, Würzburg, Germany; 6 Nuclear Medicine, Medical Faculty, University of Augsburg, Augsburg, Germany

**Keywords:** Biomarkers, Diagnostic markers, Translational research, Medical research, Preclinical research

## Abstract

We aimed to investigate the image quality of the U-SPECT5/CT E-Class a micro single-photon emission computed tomography (SPECT) system with two large stationary detectors for visualization of rat hearts and bones using clinically available ^99m^Tc-labelled tracers. Sensitivity, spatial resolution, uniformity and contrast-to-noise ratio (CNR) of the small-animal SPECT scanner were investigated in phantom studies using an ultra-high-resolution rat and mouse multi-pinhole collimator (UHR-RM). Point source, hot-rod, and uniform phantoms with ^99m^Tc-solution were scanned for high-count performance assessment and count levels equal to animal scans, respectively. Reconstruction was performed using the similarity-regulated ordered-subsets expectation maximization (SROSEM) algorithm with Gaussian smoothing. Rats were injected with ~ 100 MBq [^99m^Tc]Tc-MIBI or ~ 150 MBq [^99m^Tc]Tc-HMDP and received multi-frame micro-SPECT imaging after tracer distribution. Animal scans were reconstructed for three different acquisition times and post-processed with different sized Gaussian filters. Following reconstruction, CNR was calculated and image quality evaluated by three independent readers on a five-point scale from 1 = “very poor” to 5 = “very good”. Point source sensitivity was 567 cps/MBq and radioactive rods as small as 1.2 mm were resolved with the UHR-RM collimator. Collimator-dependent uniformity was 55.5%. Phantom CNR improved with increasing rod size, filter size and activity concentration. Left ventricle and bone structures were successfully visualized in rat experiments. Image quality was strongly affected by the extent of post-filtering, whereas scan time did not have substantial influence on visual assessment. Good image quality was achieved for resolution range greater than 1.8 mm in bone and 2.8 mm in heart. The recently introduced small animal SPECT system with two stationary detectors and UHR-RM collimator is capable to provide excellent image quality in heart and bone scans in a rat using standardized reconstruction parameters and appropriate post-filtering. However, there are still challenges in achieving maximum system resolution in the sub-millimeter range with in vivo settings under limited injection dose and acquisition time.

## Introduction

Preclinical single-photon emission computed tomography (SPECT) imaging is an evolving field full of challenges. The introduction of pinhole collimation opened up new applications for small-animal SPECT imaging^1–3^ and with the development of multi-pinhole collimation, high spatial resolution in the sub-millimeter range with acceptable sensitivity was possible^3–6^. Combining sub-millimeter precision imaging with a plethora of easily accessible radioisotopes and the option of detecting multiple radioisotopes simultaneously set SPECT apart from its competitor positron emission tomography (PET)^7^. Further, the use of stationary detectors covering 360° of a fixed field of view (FOV) enables dynamic studies with increased precision^8,9^, while reducing mechanical issues and complex system maintenance compared to a setup with moving parts^10^. The previous generations of these ultra-high-resolution SPECT systems usually have three large stationary detectors in a triangular configuration^10,11^. For this work, however, we assessed the image quality of a cost-efficient scanner that omits the bottom detector.

High spatial resolution can improve the diagnostic value of SPECT scans if sufficient image quality is achieved, which is particularly affected by the choice of collimator^12^, reconstruction algorithm^5,13^, post-reconstruction filter^13^ and injection dose^14^. Hence, we evaluated the influence of acquisition time and Gaussian post-filtering, using a novel iterative reconstruction algorithm with similarity-regulated ordered-subsets expectation maximization (SROSEM), that enables constant reconstruction parameters for a wide range of activity concentrations^15^.

Basic and translational researchers have been mainly using small-animal models such as mice or rats to reduce housing and maintenance costs, to explore pathophysiology and to develop new drugs. Rats have certain potential advantages over mice^16^. It is easier to develop suitable invasive devices for surgical procedures and hemodynamic measurements and offers larger tissue mass for histological and biological analyses. Furthermore, recent transfer of functional genomics technology into rats reemphasizes the potential of rat models^16^. For animal SPECT imaging, although bigger size of the organs is an advantage of rats over mice, the higher soft tissue attenuation and scattering, as well as requirement of larger bore and transaxial size, might have negative impact on precise imaging.

This study aims to examine the preclinical applicability and image quality of the recently introduced micro-SPECT system for rat imaging under in vivo conditions using [^99m^Tc]Tc-MIBI^17^ and [^99m^Tc]Tc-HMDP^18,19^ with a pre-set SROSEM reconstruction algorithm and Gaussian post-filtering.

## Materials and methods

### System description

**Figure 1:**
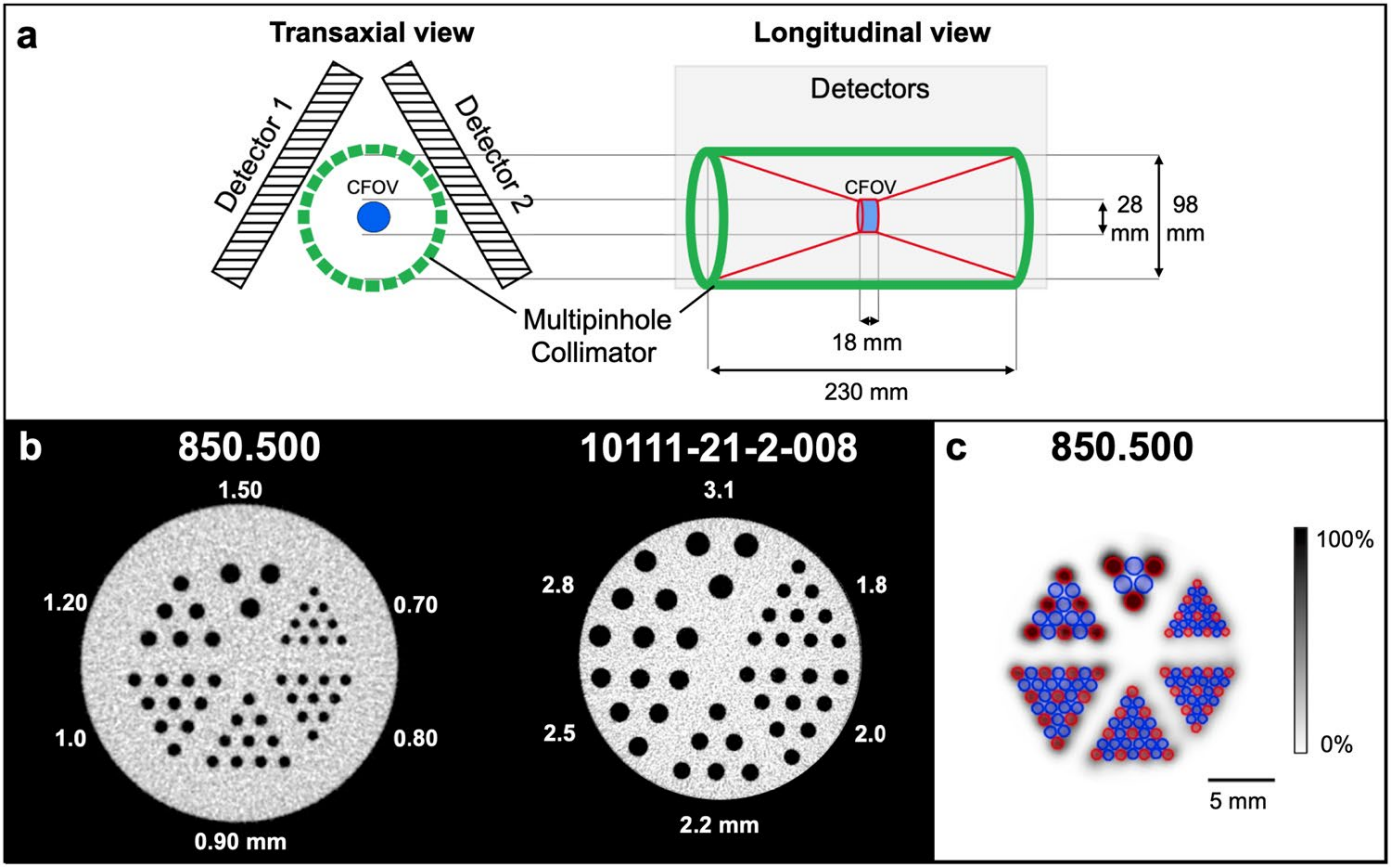
Scanner construction, mini Derenzo phantoms and contrast-to-noise ratio method. (**a**) Illustration of the arrangement of the U-SPECT5-E with two stationary detectors in two axes. Each detector, illustrated here as a square, has a surface of 472 mm × 595 mm and contains NaI(Tl) crystals with a thickness of 9.5 mm. The bore diameter of the UHR-RM collimator is 98 mm. Center field-of-view (CFOV) measures 28 mm in diameter and 18 mm in length. Scan volume can be manually adjusted to a maximum length of 230 mm. (**b**) Transaxial view of CT images of the mini Derenzo phantoms 850.500 and 10111-21-2-008 with their respective rod sizes. The shown phantoms were used to determine the maximum resolution and contrast-to-noise ratio (CNR) for various count levels. Based on CT images, the templates for CNR analysis were created. (**c**) SPECT image of the phantom 850.500 with the template for CNR analysis consisting of radioactive (red) and non-radioactive (blue) regions of interest (ROIs). ROIs have 0.9 times the diameter of corresponding rod sizes, length is 6.0 mm. This example SPECT image has a slice thickness of 6.0 mm and was taken with 285.22 MBq/mL, 300 s time per bed position and 9 bed positions.

U-SPECT5/CT E-Class (referred to as “U-SPECT5-E”; MILabs, Utrecht, The Netherlands) is an ultra-high-resolution SPECT system for preclinical imaging of small- to medium-sized animals. While the scanner architecture is based on previous generations of micro-SPECT systems (U-SPECT-II; U-SPECT^+^; MILabs)^10,11^, the conventional U-SPECT5 system features three stationary detectors with a size of 472 mm × 595 mm and 9.5 mm thick thallium-doped sodium iodide (NaI(Tl)) scintillation crystals arranged in a triangular format around the FOV. Striving for cost effectiveness, the U-SPECT5-E is built without the bottom detector as illustrated in Fig. 1a.

A centrally located collimator with multi-pinhole configuration allows the acquisition of SPECT images in a spiral step mode using the XYZ stage^20^.

In this study, an ultra-high resolution rat/mouse (UHR-RM) collimator made of tungsten was used with 75 pinholes (diameter of 1.0 mm) in 5 rows, all pointing towards a center FOV of 28 mm diameter and 18 mm length. Due to the missing bottom detector only the upper 50 pinholes contribute to the imaging process.

### Data acquisition and processing

All images were acquired in list-mode and reconstructed with the SROSEM algorithm. For simplifying the reconstruction process, we used four iterations with 128 subsets and a voxel size of 0.4 mm^3^, subsequently applying Gaussian post-filtering as advised by Vaissier et al.^15^. This approach showed no perceptible decrease in image quality compared to the reference standard of maximum likelihood expectation maximization (MLEM)^21^.

For scatter correction, we applied the triple energy window method^22^ with a photopeak window of 126 keV to 154 keV, a lower background window of 120.4 keV to 126 keV and an upper one of 154 keV to 159.6 keV. The obtained SPECT images were transferred to a workstation for further Gaussian post-filtering and analysis using AMIDE (version 1.0.5 for MacOS; open source)^23^.

### Performance measurements

Sensitivity was examined by using a point source with a ^99m^Tc-solution of 2.7 MBq. Scan time was 5 min with 1 bed position (BP). Calculation was based on the National Electrical Manufacturers Association (NEMA)^24^ with ${R}_{i}$ representing the detected photopeak countrate and ${A}_{cal}$ standing for the total activity of the point source determined by a dose calibrator (ISOMED 2010, NUVIA Instruments, Dresden, Germany).

$$ Sensitivity = \frac{{R_{i} }}{{A_{cal} }} $$

Figure 1b shows the mini Derenzo hot-rod phantom (850.500) that was used to investigate the spatial resolution. It was filled with a ^99m^Tc-solution with an activity concentration of 285.2 MBq/mL. Scan time was 45 min with 9 BP. In the visual analysis of the reconstructed data, maximum resolution was assessed as the smallest distinguishable rod size.

Uniformity was investigated by using the cylindrical container of the mini Derenzo phantom. The filling volume was 10.6 mL with a ^99m^Tc-solution of 315.0 MBq and the scan time was 45 min with 9 BP. Gaussian post-filtering was applied with a full width at half maximum (FWHM) of 1.2 mm representing the maximum resolution. For calculation, a cylindrical region of interest (ROI) was placed centrally in the phantom, measuring 18 mm in diameter and 10 mm in length. Collimator-dependent system uniformity was calculated as recommended by NEMA^24^.

$$ Uniformity \left( \% \right) = 100 \times \frac{Max\;count - Min\; count}{{Max\;count + Min \;count}} $$

### Phantom image quality

To evaluate the in vitro image quality, a contrast-to-noise ratio (CNR) analysis was carried out by using two mini Derenzo phantoms. This technique, as shown in Fig. 1c, was described initially by Walker et al.^25^. Using a high-resolution computed tomography (CT) image as a template, ROIs of 6 mm length were placed in the center of the rods. All ROIs have a diameter of 0.9 times the size of the respective radioactive rod. In addition, ROIs of the same size were placed in the non-radioactive regions in-between two radioactive rods.

The contrast ${C}_{d}$ was defined as:

$$ C_{d} = \frac{{\overline{{R_{d} }} - \overline{{B_{d} }} }}{{\overline{{R_{d} }} }} $$

$\stackrel{-}{{R}_{d}}$ is the mean value of all radioactive ROIs and $\stackrel{-}{{B}_{d}}$ is the mean value of all non-radioactive ROIs for the rod size $d$.

The noise ${N}_{d}$ was defined as:

$$ N_{d} = \frac{{\sqrt {\sigma_{{R_{d} }}^{2} + \sigma_{{B_{d} }}^{2} } }}{{\overline{{ROIs_{d} }} }} $$

${\sigma }_{{R}_{d}}$ and ${\sigma }_{{B}_{d}}$ are the standard deviations in radioactive and non-radioactive ROIs, while $\stackrel{-}{{ROIs}_{d}}$ is the mean value of all ROIs of a rod size $d$, no matter if they are radioactive or non-radioactive.

These two formulas are used to calculate the CNR:

$$ CNR_{d} = \frac{{C_{d} }}{{N_{d} }} $$

This image quality analysis was performed for the smaller 850.500 and the larger 10111-21-2-008 phantom with a rod size range of 0.7–1.5 mm and 1.8–3.1 mm, respectively. In addition to high-count performance measurements with ~ 290.0 MBq/mL, low-count measurements were also conducted (~ 1.0 MBq/mL, ~ 0.5 MBq/mL, ~ 0.1 MBq/mL). To minimize the loss of resolution, images were optimized by a Gaussian post-filter for each rod size. The kernel size of the filter always corresponded to FWHM = rod size.

### Animal studies

Animal protocols were approved by the local Animal Care and Use Committee (Regierung von Unterfranken, Germany) and conducted according to the Guide for the Care and Use of Laboratory Animals^26^.

### [^99m^Tc]Tc-HMDP bone rat imaging

One healthy female Wistar rat (Charles River Laboratories, Sulzfeld, Germany) with 217.0 g body weight was injected 154.86 MBq of [^99m^Tc]Tc-HMDP^18,19^ via tail vein. Acquisition started at 1 h post-injection with a total scan time of 90 min containing 18 5-min-frames with 20 s time per bed position (TPB) and 15 BP. During acquisition, the rat underwent an inhalation anesthesia (2.0% isoflurane, 1.5 L O_2_/min).

**Figure 2:**
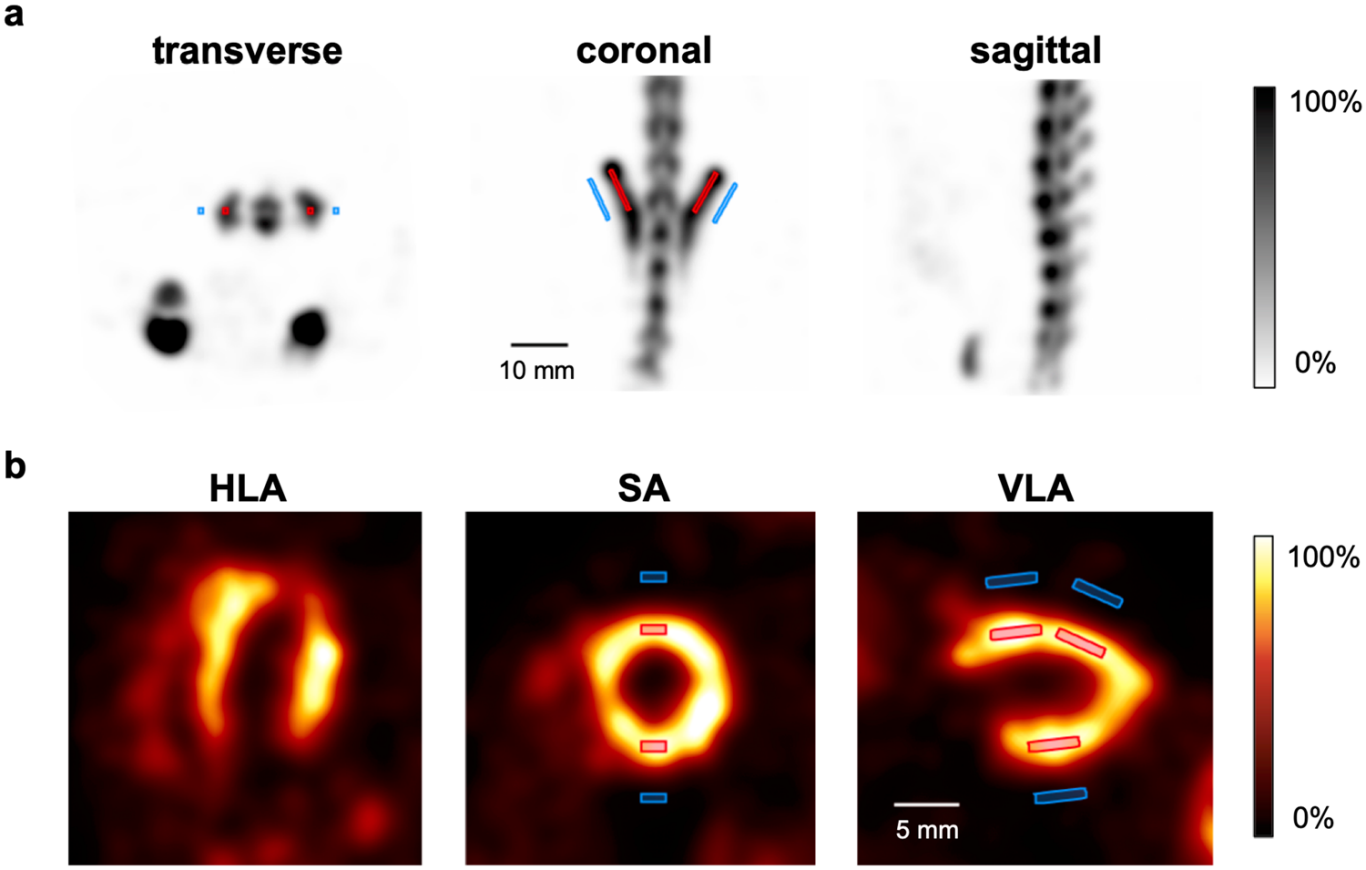
In vivo contrast-to-noise ratio method. (**a**) [^99m^Tc]Tc-HMDP SPECT image of the lower spine and pelvis region of a healthy rat from transverse, coronal and sagittal perspective. Illustration of the in vivo contrast-to-noise ratio (CNR) analysis for the focused bone scan by using radioactive (red) and non-radioactive (blue) regions of interest (ROIs) with a size of 0.8 × 8.0 × 1.2 mm^3^ each. The rat was injected with 154.9 MBq [^99m^Tc]Tc-HMDP and scanned 60 min post injection for 90 min total scan time. (**b**) [^99m^Tc]Tc-MIBI SPECT image of a healthy rat cropped and rotated to the horizontal long axis (HLA), short axis (SA) and vertical long axis (VLA) view of the heart. The ROIs for CNR analysis in the heart had a size of 4.0 × 0.8 × 2.0 mm^3^ each. The rat was injected with 108.5 MBq [^99m^Tc]Tc-MIBI and scanned 25 min post injection for 60 min total scan time. Reconstructed images were filtered with a Gaussian filter (full width at half maximum (FWHM) = 2.2 mm) and are shown with a slice thickness of 0.4 mm.

To determine the CNR in the reconstructed bone images, one radioactive ROI was placed centrally in the pelvic bone on each side and a corresponding non-radioactive ROI was placed in the background at 4 mm distance. All ROIs in the bone scan were box-shaped and had a size of 0.8 × 8.0 × 1.2 mm^3^ (Fig. 2a). This analysis was applied to the focused bone scan with reconstructed frame lengths of 5, 30 and 90 min. The three reconstructions were analyzed unfiltered and for five different Gaussian post-filters (1.2, 1.8, 2.2, 2.8 and 3.5 mm).

### [^99m^Tc]Tc-MIBI cardiac rat imaging

Another healthy female Wistar rat (Charles River Laboratories), weighing 231.5 g, was injected 108.51 MBq of [^99m^Tc]Tc-MIBI^17^ into the tail vein. Twenty-five minutes after injection, a total-body scan was performed with an acquisition time of 60 min, divided into six 10-min-frames (TPB 15 s, BP 40). While scanning, an inhalation anesthesia (2.0% isoflurane, 1.5 L O_2_/min) was carried out. By using the heart uptake of [^99m^Tc]Tc-MIBI described in the literature with 1.71 ± 0.63%ID/g for the myocardium of the rat^27^, we calculated the expected activity in the rat heart to match it with the phantom studies, taking into account the different TPB.

For the reconstructed data, three radioactive ROIs were placed centrally in the wall of the left ventricle and three non-radioactive ROIs were each positioned 4 mm away from the corresponding radioactive ROI in the background outside of the heart. All ROIs in the heart scan were box-shaped and had a size of 4.0 × 0.8 × 2.0 mm^3^ (Fig. 2b). This analysis was applied to the whole-body heart scan with frame lengths of 10, 30 and 60 min. Again, all three images were analyzed unfiltered and for five different Gaussian post-filters (1.2, 2.2, 2.8, 3.5 and 4.0 mm).

### In vivo image quality

#### Contrast-to-noise ratio

The CNR was calculated in the same way as described above for the mini Derenzo phantoms. However, instead of using the ROIs for one specific rod diameter $d$, the ROIs for the respective animal scan were employed.

#### Visual image quality assessment

All images were sent to three independent readers for visual assessment of image quality as vertical long axis view (heart) and coronal view (bone). The images were doubled and randomized. Observers were blinded to the acquisition and post-processing protocol and were asked to rate the overall image quality on a five-point scale (1 = “very poor”, 2 = “poor”, 3 = “moderate”, 4 = “good”, 5 = “very good”).

#### Statistics

Statistical analysis was carried out with specialized software (SPSS Statistics Version 27 for MacOS, IBM, Amonk, New York, USA). Kolmogorov–Smirnov tests were applied to assess normal distribution of continuous variables. Categorical variables are presented as percentages, frequencies and median values with interquartile range (IQR), e.g. for image quality scale results.

## Results

### Performance measurements

**Figure 3:**
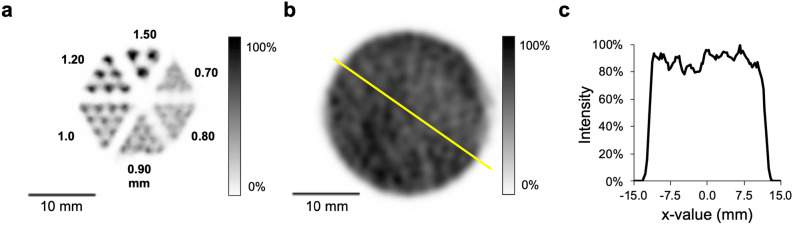
Resolution and uniformity. (**a**) shows a representative SPECT image of the hot-rod phantom (850.500), filled with 285.2 MBq/mL ^99m^Tc-solution to analyze maximum resolution. Minimal discriminable rod size was 1.2 mm. Uniformity phantom (**b**), filled homogenously with 29.7 MBq/mL ^99m^Tc-solution and line profile (**c**) corresponding to the yellow line. Gaussian post-filter equals maximum resolution of 1.2 mm.

Point source sensitivity examined with technetium-99m for the UHR-RM collimator was 567 cps/MBq (0.057%). In visual analysis of the hot-rod phantoms’ rod sections, a minimum diameter of 1.2 mm could be discriminated, what was assumed to represent the maximum resolution (Fig. 3a). Uniformity for the UHR-RM collimator was 55.5% in accordance with the NEMA protocol. Figure 3b,c illustrate reconstructed image and corresponding line profile.

### In vitro image quality

**Figure 4:**
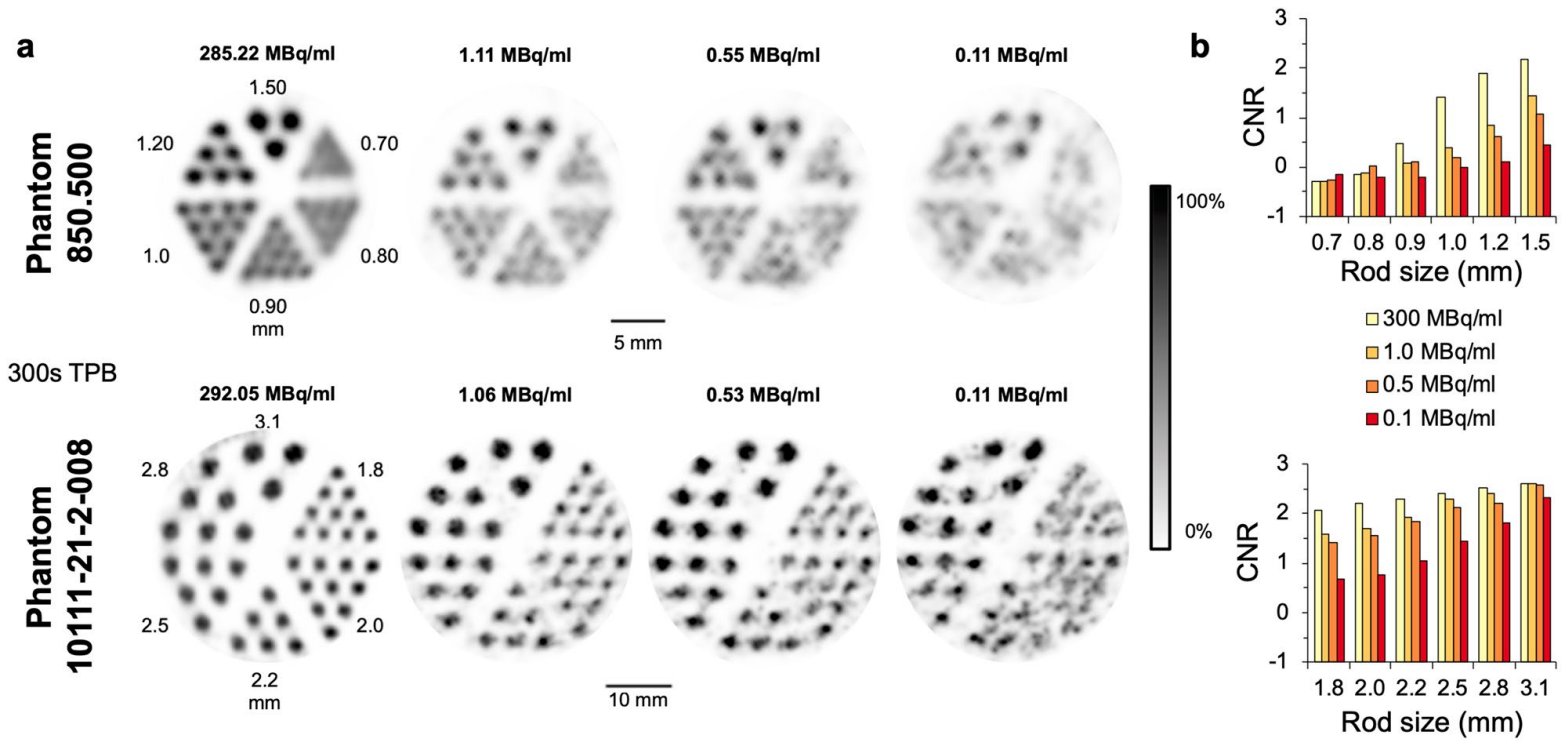
Hot-rod phantom scans and corresponding contrast-to-noise ratios. (**a**) SPECT images of phantoms 850.500 and 10111-21-2-008 with one high and three low activity ^99m^Tc-solutions for a 300 s time per bed position (TPB) scan (9 bed positions for the 850.500, 6 bed positions for the 10111-21-2-008). Intensity scale was adapted for each image to enhance contrast. Results are shown in transaxial view with slice thickness of 6.0 mm. Gaussian post-filter of full width at half maximum (FWHM) = 0.7 mm was applied. (**b**) Bar charts display corresponding contrast-to-noise ratio of phantom scans optimized for each rod size by Gaussian post-filter of FWHM = rod size.

Figure 4a provides an illustration of the phantom images used for CNR analysis. For the lower activity an increased image noise is visible, while contrast remains fairly constant.

Figure 4b shows the dependency of the CNR on the investigated rod size for four different activity concentrations. While measurements in rods smaller than 1.2 mm mostly resulted in very low, partly negative CNR values (− 0.31 to 0.48), only 300 MBq/mL achieved a higher value below the maximum resolution (1.41 at 1.0 mm). CNR of 1.50 was achieved for 300 MBq/mL, 1.0 MBq/mL, 0.5 MBq/mL and 0.1 MBq/mL at rod sizes of 1.2 mm, 1.8 mm, 2.0 mm and 2.8 mm, respectively. For all activity concentrations, CNR improved continuously with increasing rod size and Gaussian filtering.

### In vivo image quality

#### [^99m^Tc]Tc-MIBI cardiac rat imaging

**Figure 5:**
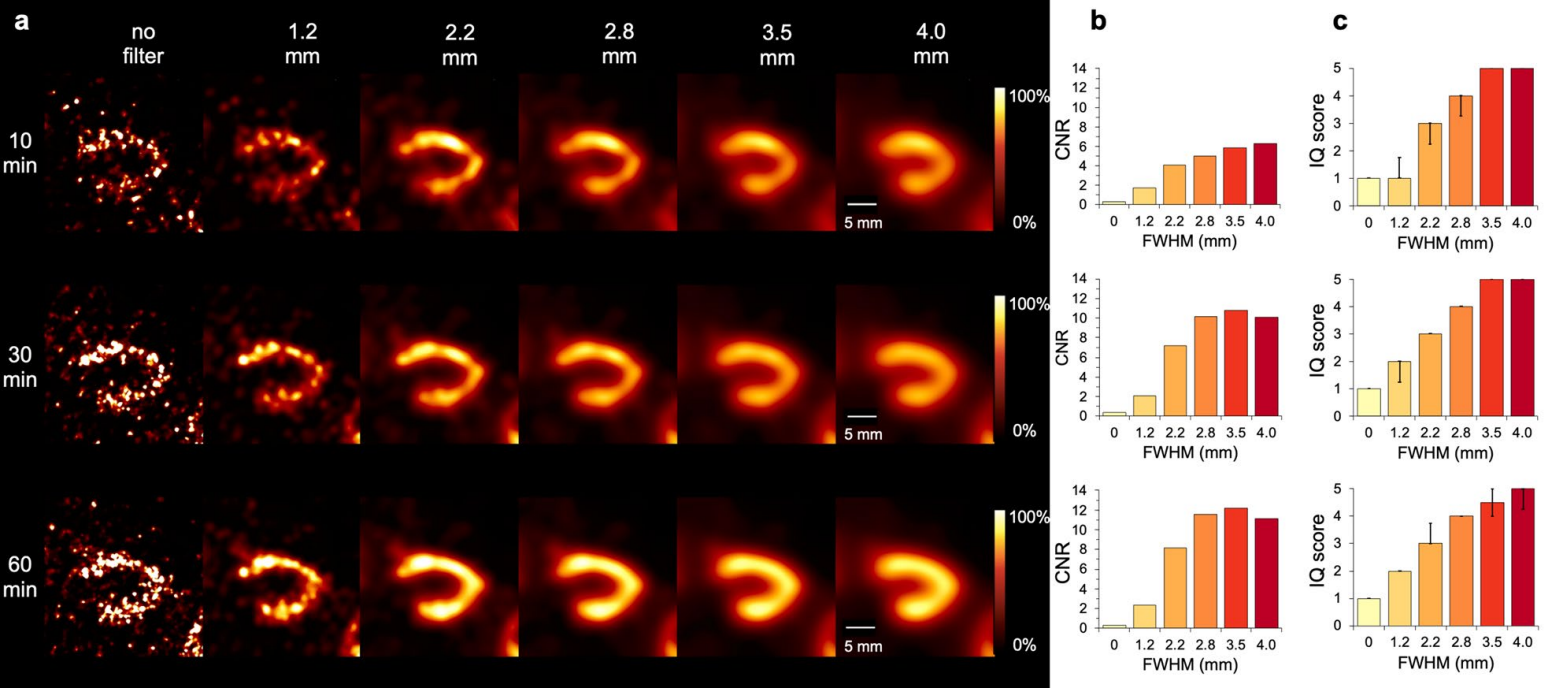
Heart scan assessment. (**a**) Myocardial perfusion SPECT with three different scan times and six different Gaussian post-filters (FWHM = full width at half maximum). Vertical long axis perspective images have a slice thickness of 0.4 mm with adjusted color scale. [^99m^Tc]Tc-MIBI injection dose was 108.5 MBq, and the scan was performed 25 min after tracer injection into tail vein for 60-min acquisition (40 bed positions, 15 s time per bed position). Image quality analysis is based on images reconstructed from extracted scan data of 10 min, 30 min and 60 min. (**b**) Results of corresponding contrast-to-noise ratio calculations. (**c**) Image quality scores given by three independent readers displayed as median with interquartile range in bar charts.

SPECT images of the investigated healthy rat heart for different acquisition times (10, 30 and 60 min) and various post-reconstruction Gaussian filters (FWHM of 1.2, 2.2, 2.8, 3.5 and 4.0 mm) are displayed in vertical long axis view in Fig. 5a. The images from all three perspectives (horizontal long axis, short axis, vertical long axis) are shown in supplemental Fig. 1. No artifacts were detected. Left ventricle of the heart is clearly visualized as the myocardium shows sufficient uptake of [^99m^Tc]Tc-MIBI. Images of each scan time without any filtering result in poor image quality and high noise. Gaussian post-filtering clearly improved image quality for each scan.

The results for the in vivo contrast-to-noise ratio and image quality assessment of the healthy rat heart are summarized in Fig. 5b,c. Increasing the FWHM up to 3.5 mm of the Gaussian filtering results in improved CNR. The highest values were achieved for the 10-min scan with 4.0 mm and for the 30-min and 60-min scan with 3.5 mm kernel size, respectively (Fig. 5b). Comparing the three different acquisition times and therefore different count levels, peak values for CNR increased substantially from 10 min (6.3) to 60 min (12.2).

**Figure 6:**
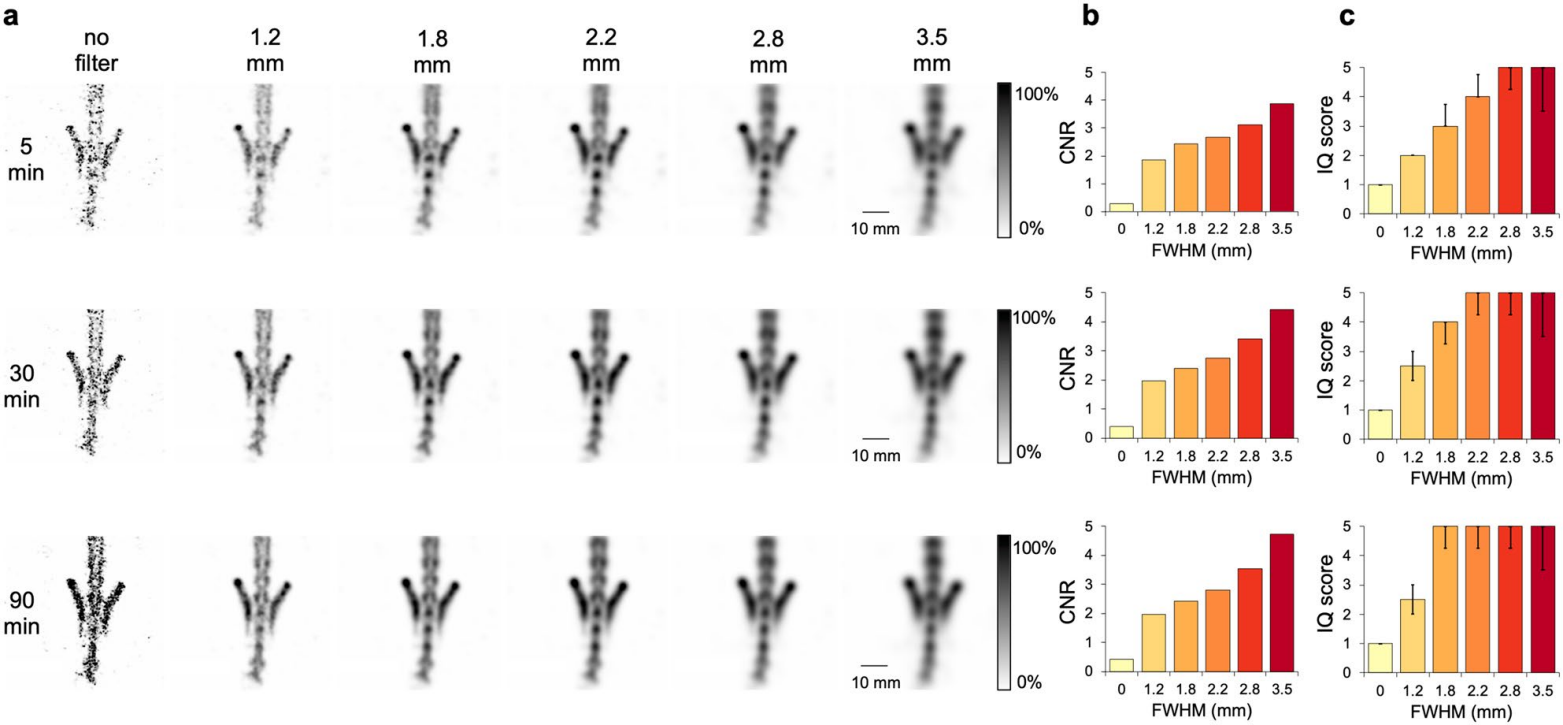
Bone scan assessment. (**a**) Bone SPECT images with three different scan times and six different Gaussian post-filters (FWHM = full width at half maximum). The scan was focused on pelvis and lower spine of a healthy rat, the top-view perspective is shown with a slice thickness of 0.4 mm and adjusted scaling for good contrast. [^99m^Tc]Tc-HMDP injection dose was 154.9 MBq, and the scan was performed 60 min after tracer injection into tail vein for 90 min acquisition (15 bed positions, 20 s time per bed position). Image quality analysis is based on images reconstructed from extracted scan data of 5 min, 30 min and 90 min. (**b**) Results of corresponding contrast-to-noise ratio calculations. (**c**) Image quality scores given by three independent readers displayed as median with interquartile range in bar charts.

Peak image quality scores for the investigated scan times were 5.00 (IQR = 0.00) for 10 min and 30 min with FWHM of 3.5 and 4.0 mm, 5.00 (IQR = 0.75) for 60 min with FWHM of 4.0 mm (Fig. 6c). All images without filtering received the lowest rating “very poor”. FWHM = 1.2 mm scored in the range of “very poor” to “moderate”, 2.2 mm “poor” to “good” and 2.8 mm “moderate” to “very good”.

#### [^99m^Tc]Tc-HMDP bone rat imaging

Reconstructed images of the healthy rat’s lower spine and pelvis region with various Gaussian filters (FWHM = 1.2, 1.8, 2.2, 2.8 and 3.5 mm) and three different scan times (5, 30 and 90 min) are shown from the coronal view in Fig. 6a. The images from all three perspectives (transverse, coronal, sagittal) are shown in supplemental Fig. 2. No artifacts were detected. Differentiation of vertebra and intervertebral discs was feasible, the pelvic bones were visualized in detail. Images without any filtering appeared noisy and provided poor detail. Nevertheless, higher count levels and longer acquisition time led to an overall improvement of image quality. By increasing the post-reconstruction Gaussian filter, image quality was further enhanced. High filtering resulted in homogenous and smooth images but provided less detail and seemed overly blurred.

Figure 6b depicts results of the in vivo CNR analysis. Irrespective of acquisition time, Gaussian filtering of 3.5 mm was associated with the highest CNR values. However, no clear difference in absolute CNR values could be determined by extending the acquisition time.

Figure 6c plots the image quality scores of images shown in Fig. 6a as a function of Gaussian kernel size. Peak image quality scores of 5.00 for the investigated scan times were achieved for 5 min and FWHM of 2.8 and 3.5 mm, for 30 min and FWHM of 2.2, 2.8 and 3.5 mm and for 90 min and FWHM ≥ 1.8 mm. Quality of unfiltered images scored 1.00 (IQR = 0.00) for every acquisition time by all readers. Ratings for kernel size 1.2 mm ranged from “poor” to “moderate”. Image quality for FWHM of 1.8 mm was considered “moderate” to “good”. The images filtered with a kernel size of 2.2, 2.8 and 3.5 mm were all rated “moderate” to “very good”.

## Discussion

We investigated to what extent the recently introduced two-detector U-SPECT5-E with UHR-RM collimator and the novel SROSEM algorithm is suitable for ultra-high-resolution rat imaging in a realistic in vivo setting.

In the performance evaluation, the SPECT system achieved a sensitivity of 567 cps/MBq, a resolution of 1.2 mm and a uniformity of 55.5%. This was comparable to the previous generation with three detectors and a similar multi-pinhole rat collimator, which achieved a sensitivity of 700 cps/MBq and a resolution of 0.8 mm. It should be noted that an activity concentration of 600 MBq/mL was used^10^. No direct comparison was made between the two-detector system and the three-detector system regarding the rat imaging capabilities. Such comparison might reveal the impact of the missing lower detector in more detail. Boisson et al. evaluated a system with rotating detectors and three different rat collimators with maximum resolutions in the range of 1.1–2.0 mm but sensitivities below 300 cps/MBq. They only used either one or three pinholes^28^. For another established system with four detectors, three rat collimators were reported with maximum resolutions in the range of 1.1–1.9 mm and sensitivities of up to more than 2000 cps/MBq^29^, but more detailed methodological information is lacking and the recently published study on the new generation of the system contains mainly information on the mouse collimators^30^. However, it should be noted that the activity concentrations in these performance evaluations using phantoms are much higher than the activity concentrations under realistic in vivo conditions.

In the in vitro CNR analysis, values were considerably lower for small rod sizes and lower activity concentrations and increased with larger rod size and post-filtering. Similar observations were made for pinhole PET by Walker et al.^25^. The Gaussian post-filtering results in a maximum resolution (FWHM) limited by the kernel size of the filter. The CNR values in phantom measurements (Fig. 4) were substantially lower at low activities compared to high activities. To achieve a CNR similar to values in rods of 1.2 mm at 300 MBq/mL, larger rods and filters were required in the in vivo study count range. Hence, reasonable CNR values were achieved for 1.0 MBq/mL with ≥ 1.8 mm, for 0.5 MBq/mL with ≥ 2.0 mm and for 0.1 MBq/mL with ≥ 2.8 mm rod size.

Multi-observer analysis of the longest in vivo scans (60 and 90 min) gives a count range comparable to approximately 1.0 MBq/mL in phantom studies. Good CNR and image quality scores were found for the SPECT images with Gaussian kernels bigger than 1.8 mm in bone and 2.8 mm in the rat heart. This implies that the maximum resolution that can realistically be achieved for in vivo imaging is slightly lower than the system resolution of 1.2 mm.

Analysis of in vivo heart studies revealed a substantial increase in CNR between 10 and 30 min, while extension of acquisition time to 60 min resulted in only a minor increase. A similar tendency was seen in the visual image analysis, where the scan time did not lead to a relevant image quality increase and the longest scan time of 60 min even resulted in inferior image quality ratings. This loss of image quality could be the result of slightly more intensive scaling of the 60 min images. In contrast to acquisition time, the choice of filter size had considerably more effect on subjective ratings. While 4.0 mm led to the best results for short scan times in the heart, the best CNR value could be achieved with a longer scan time and a filter of 3.5 mm. The filters of 3.5 mm or 4.0 mm also provided the best subjective image quality for professional readers. Nonetheless, even a filter of 2.2 mm delivered acceptable image quality and can be suitable if the additional resolution is required for certain imaging tasks. Mizutani et al. also investigated the influence of injection dose and post-filtering on image quality in the rat heart. A SPECT system with cadmium-zinc telluride detectors and two different five-pinhole collimators was used (sensitivity = 321 cps/MBq, 139 cps/MBq; resolution = 1.5 mm, 1.2 mm). The evaluation by two readers showed an increase in image quality by higher injection dose (25–200 MBq) and stronger filters (no filter; FWHM = 1.5 mm, 2.5 mm)^14^. The injection dose had more impact on the image quality score than we found for the scan time, but the results are still largely consistent with ours.

In the case of bone imaging, stronger filtering increased CNR without restriction, although the anatomical structures appeared blurry throughout. The bone structure in the area of the lumbar spine and pelvis is very precise and complex, which might lead to a supposedly high noise level in the calculation. On the other hand, observer analysis showed no considerable increase between the different scan times of 5, 30 and 90 min, while increasing the Gaussian filter from 1.2 mm to 1.8 mm leads to a noticeably higher image quality score. The positive effect of post-filtering on CNR and image quality can most likely be attributed to the improvements in uniformity and noise^13^. The study is limited by the use of only one rat per tracer. A certain inter-animal variance could be expected, since we focused on the impact of scan time and post filter on the image quality and not the quantitative capabilities, we decided to use only two examples with well-established tracers for reasons of good animal practice.

## Conclusion

We analyzed the performance of a recently introduced ultra-high-resolution micro-SPECT system with two stationary detectors for preclinical rat imaging. Providing good image quality with a multi-pinhole UHR-RM collimator, the scanner is suitable for heart and bone scans using standardized reconstruction parameters and appropriate post-filtering. Although the system demonstrates excellent performance in rat imaging as compared to conventional systems, there are still challenges to achieve sub-millimeter system resolution in rats where there are safety limits on injection dose and acquisition time.

## Supplementary information

Supplementary Information

## Data Availability

The datasets generated and/or analyzed during the current study are available from the corresponding author on reasonable request.
