# Supplementary material for: Capabilities of multi-pinhole SPECT with two stationary detectors for in vivo rat imaging
Source: Sci Rep. 2020 Oct 29;10:18616. doi: 10.1038/s41598-020-75696-0 (PMC7596477; doi:10.1038/s41598-020-75696-0)
Supplement: Supplementary file 1 — Supplementary Information [file 41598_2020_75696_MOESM1_ESM.pdf]

Supplementary Information

Capabilities of Multi-Pinhole SPECT with Two Stationary Detectors for In Vivo Rat Imaging

Jan P. Janssen[1,2#], Jan V. Hoffmann[1,2#], Takayuki Kanno[2,3], Naoko Nose[4], Jan-Peter Grunz[5], Masahisa Onoguchi[3], Xinyu Chen[2,6], Constantin Lapa[6], Andreas K. Buck[1], Takahiro Higuchi[1,2,4*]

[1]*Department of Nuclear Medicine, University Hospital Würzburg, Würzburg, Germany*
[2]*Comprehensive Heart Failure Centre, University Hospital Würzburg, Würzburg, Germany*
[3]*Department of Quantum Medical Technology, Graduate School of Medical Sciences, Kanazawa University, Kanazawa, Japan*
[4]*Graduate School of Medicine, Dentistry and Pharmaceutical Sciences, Okayama University, Okayama, Japan*
[5]*Department of Diagnostic and Interventional Radiology, University Hospital Würzburg, Würzburg, Germany.*
[6]*Nuclear Medicine, Medical Faculty, University of Augsburg, Augsburg, Germany.*

***Corresponding Author:**

Prof. Takahiro Higuchi, MD, PhD
Department of Nuclear Medicine, University Hospital Würzburg,
Oberdürrbacher Strasse 6, 97080 Würzburg, Germany
Telephone: +49(931)201-35455, Fax: +49(931)201-635000, Email: higuchi_t@ukw.de

[#]First Authors:

Jan Paul Janssen (MD student)
Department of Nuclear Medicine, University Hospital Würzburg,
Oberdürrbacher Strasse 6, 97080 Würzburg, Germany
Telephone: +49(931)201-35001, Email: jan_paul.janssen@stud-mail.uni-wuerzburg.de

Jan Vincent Hoffmann (MD student)
Department of Nuclear Medicine, University Hospital Würzburg,
Oberdürrbacher Strasse 6, 97080 Würzburg, Germany
Telephone: +49(931)201-35001, Email: jan.hoffmann1@stud-mail.uni-wuerzburg.de

---

[#]These authors contributed equally to the current article.

Supplementary Figures

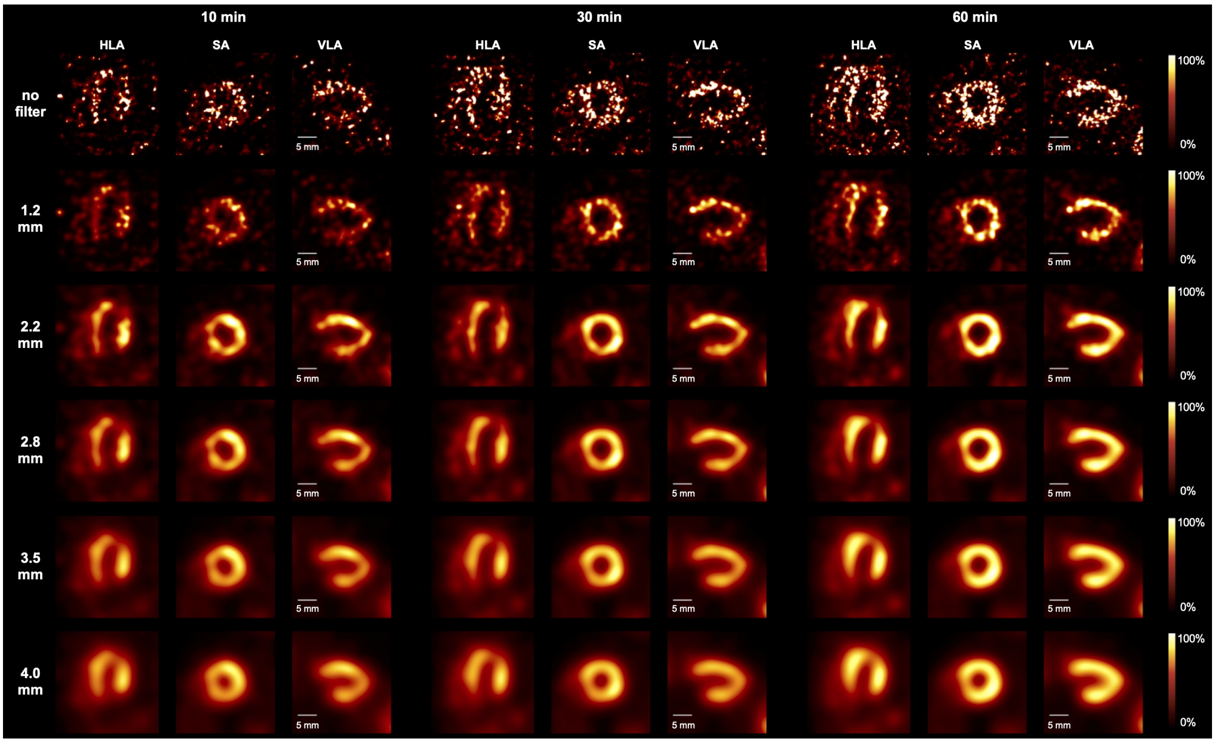

Supplementary FIGURE 1 Myocardial perfusion SPECT with three different reconstructed scan times and six different Gaussian post-filters. Whole-body scan of a healthy rat cropped to the heart region. Horizontal long axis (HLA), short axis (SA) and vertical long axis (VLA) perspective images have a slice thickness of 0.4 mm and adjusted scaling for good contrast. Injection dose was 108.5 MBq $_{99m}$Tc-MIBI and 25 min after tracer injection into tail vein the scan was performed for six 10-min frames (40 BP, 15 s TPB). Reconstructed scan times consisting of the first (10 min), the first three (30 min) and all six frames (60 min) are displayed.

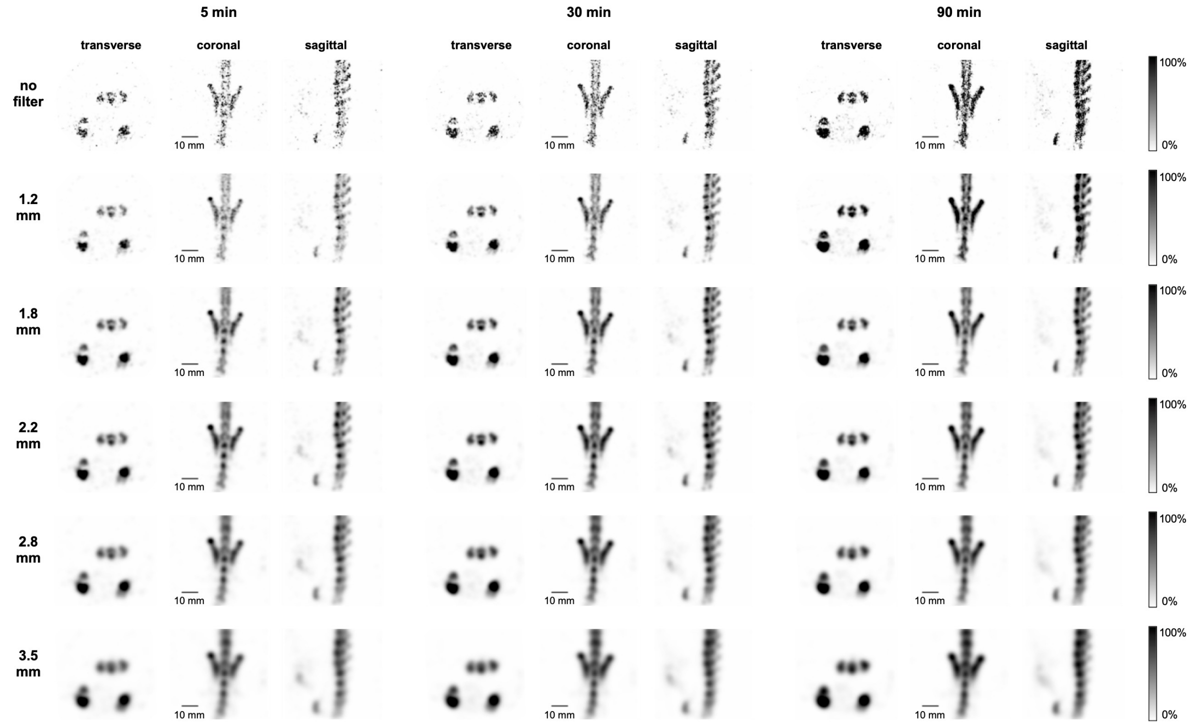

Supplementary FIGURE 2 Bone SPECT images with three different reconstructed scan times and six different Gaussian post-filters. The scan was focused on pelvis and lower spine of a healthy rat, the transverse, coronal and sagittal view is shown with a slice thickness of 0.4 mm and adjusted scaling for good contrast. Injection dose was 154.9 MBq $_{99m}$Tc-HMDP and 60 min after tracer injection into tail vein, the scan was performed for 18 5-min frames (15 BP, 20 s TPB). Reconstructed scan time consisting of the first (5 min), the first six (30 min) and all 18 frames (90 min) are displayed.
